# Takayasu arteritis as a cause of arterial hypertension. Case report and literature review

**DOI:** 10.1007/s00431-012-1674-z

**Published:** 2012-01-31

**Authors:** Elżbieta Sadurska, Renata Jawniak, Marek Majewski, Elżbieta Czekajska-Chehab

**Affiliations:** 1 Department of Pediatric Cardiology, Medical University of Lublin, ul. Chodźki 2, 20-093 Lublin, Poland; 2 Department of Pediatric Nephrology, Medical University of Lublin, Lublin, Poland; 3 Department of Radiology, Medical University of Lublin, Lublin, Poland

**Keywords:** Takayasu arteritis, Arterial hypertension, Children

## Abstract

We report a 16-year-old girl in whom Takayasu arteritis (TA) was manifested mainly by severe arterial hypertension on her right arm, which was detected during a routine examination at school. Her systolic blood pressure on the right arm was significantly higher than that on the left one. There was also a pressure difference between the right arm and legs. The pulse of the left external carotid artery and that of the left radial artery was absent. Vascular bruits over interscapular and right supra- and subclavian areas were heard on auscultation. The diagnosis of TA was confirmed by a spiral computed tomography angiography, which showed a thickened thoracic aortic wall and narrowing of its lumen. In addition, complete occlusion of the left common carotid artery and the left subclavian artery was observed. *Conclusion:* The rarity of the disorder and the heterogeneous nature of its clinical manifestation predispose to a late diagnosis and delayed treatment. Our report highlights the fact that the condition can and does occur in a pediatric population in Europe and hence must be considered in patients presenting with suggestive symptoms and signs, especially in young patients with unexplained hypertension. Clinical suspicion and proper imaging are crucial for the correct diagnosis and management of patients with TA. A brief review of literature completes this report.

## Introduction

Takayasu arteritis (TA), also known as aortoarteritis and pulseless disease, is a rare condition. It is a form of granulomatous arteritis, which affects large- and medium-sized arteries, primarily the aorta and its large branches as well as proximal portions of pulmonary, coronary, and renal arteries. Initially, there are mononuclear cell infiltrations in the adventitia and granulomas with Langerhans cells in the media, followed by disruption of the elastin layer and subsequent massive medial and intimal fibrosis. These lesions result in segmental stenosis, occlusion, dilatation, and aneurysmal formation in the affected vessels [21]. TA is predominantly a disease of young adults in the second and third decades of life, but it has also been reported in childhood and in adults older than 40 years [20, 28, 44]. The youngest patient described was 6 months old [26], and the oldest one was 75 years [30]. Females are more likely to be affected than males. In adults approximately 80% of patients with TA are women [44], although the female-to-male ratio varied from 9:1 in reports from Japan [27], 6.9:1 in Mexico to 1.2:1 in Israel [8]. In the pediatric population, the female preponderance is less obvious. A series of studies of TA in childhood from India and South Africa report a 2:1 female-to-male ratio [16, 46]. Although the disease has a worldwide distribution, the incidence is considered to be substantially greater in regions such as India and East Asia than Europe or North America. Unfortunately, there are no worthwhile epidemiological studies from these supposedly high incidence countries to support this assertion. According to the published reports, the worldwide incidence of TA is estimated at 2.6 cases per million population per year [44]. About 200 new cases of TA are registered annually in Japan [27]. The incidence of TA is estimated to be 2.6 persons per million population per year in Minnesota Olmsted County [17]. However, the applicability of this number to the diverse population of the USA as a whole is uncertain. The incidence in Europe overall is reported at one case per million persons per year [8]. In Sweden, the annual incidence is 1.2 per million [52], whereas in the United Kingdom it is 0.15 case per million per year [44]. The prevalence of TA in children is unknown. Kerr et al. included about 30% of pediatric patients in their study and reported an incidence in all ages of 2.6 per million [24]. Although patients with Takayasu arteritis may present with numerous clinical manifestations, arterial hypertension is the most common feature of the disease in both adults and children [10, 16, 17, 24, 46].

## Case report

A 16-year-old Caucasian girl was referred to our hospital due to elevated arterial blood pressure, which was detected by chance. Upon admission, the girl was in good general condition. The physical examination revealed normal physical and mental development. Her body weight and height were 49 kg (the10th percentile) and 161 cm (the 25th percentile), respectively, with a BMI of 18/kg/m^2^. Tanner stage III was recorded. The girl virtually never had any complaints. However, weakness and fatigability of the upper left extremity were disclosed by a detailed history. In addition, we found out that 2 years earlier, difficulties in measurement of blood pressure on her left arm had occurred. Her family history was unremarkable.

On physical examination, there was no evidence of heart failure. The heart rate was regular at 90/min. Cardiac sounds were normal. A soft (grade 2/6) systolic murmur was audible over the cardiac apex. Vascular bruits over the interscapular and right supra- and the subclavian areas were heard on auscultation. Neither the liver nor the spleen was enlarged. The muscles of the upper left extremity were slightly atrophic, and their tone was decreased. The pulse of the left external carotid artery and that of the left radial artery was absent. The femoral pulses were present and equal. On admission, a significantly elevated systolic blood pressure 192/77 mmHg on the right arm was observed (systolic blood pressure above 95th percentile for patient’s age, gender, and height, and normal diastolic blood pressure), whereas the measurement of blood pressure on the left arm was difficult but was about 90/52 mmHg. The blood pressure on right and left legs was 137/74 and 134/70 mmHg, respectively. Fundoscopic examination was normal.

**Fig. 1:**
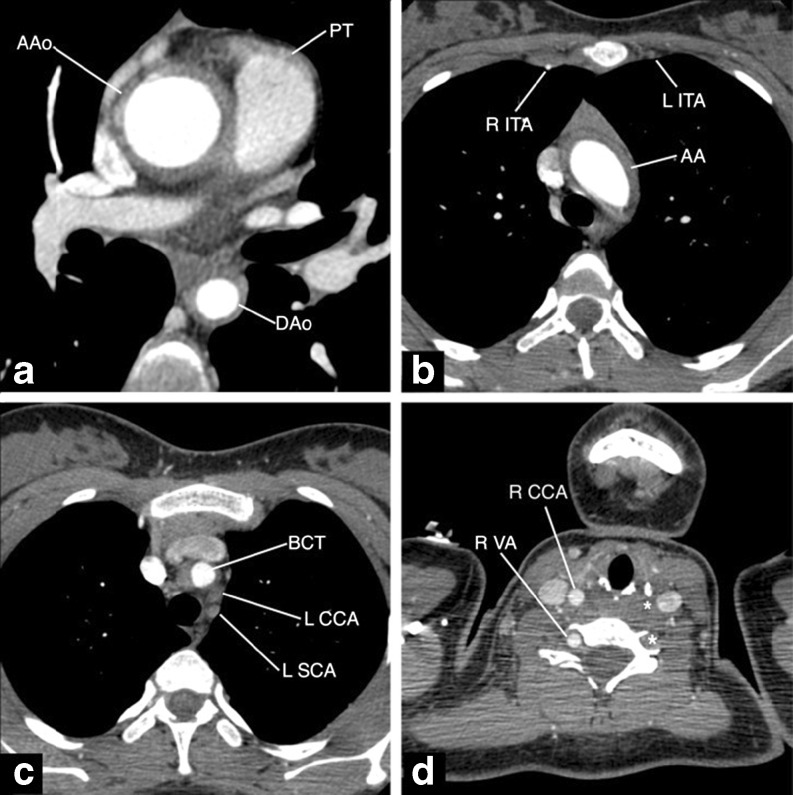
Spiral computed tomography angiography examination, cross-section slices presenting circumferential thickening of aortic wall. **a** Level of AAo and DAo with slightly thickened wall of proximal section of pulmonary trunk; **b** level of aortic arch with nonenhanced L ITA; **c** proximal sections of aortic arch branches with occluded L CCA and L SCA; **d** widening of R VA, lack of opacification of L VA and L CCA (marked with *arrows*). *AAo* ascending aorta, *AA* aortic arch, *DAo* descending aorta, *BCT* brachiocephalic trunk, *CCA* common carotid artery (*L* left, *R* right), *SCA* subclavian artery (*L* left, *R* right), *VA* vertebral artery (*L* left, *R* right), *ITA* internal thoracic artery (*L* left, *R* right), *PT* pulmonary trunk

**Fig. 2:**
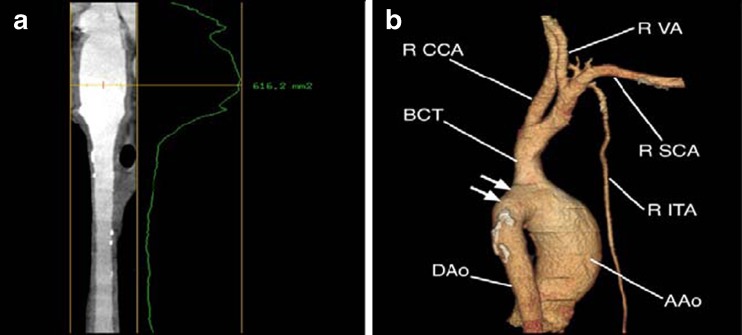
SCTA examination—secondary 2D and 3D reconstructions. **a** Vessel analysis protocol—thickened aortic wall with slightly narrowed lumen of DAo; **b** volumetric reconstruction view of branches of aortic arch with occluded L CCA and L SCA marked with *arrows*

Laboratory findings showed an elevated erythrocyte sedimentation rate (ESR) of 46 mm/h (normal value <20 mm/1 h) and serum C-reactive protein level of 1.13 mg/dl (normal value <0.5 mg/dl). In addition, anemia (hemoglobin level of 5.75 mmol/l) and an increased level of serum immunoglobulin G were detected. The rest of the laboratory investigations, including serum creatinine, electrolytes, autoantibodies, and urinalysis, were normal, as well as the ECG and chest X-ray. The tuberculin test was negative. The renal color Doppler ultrasound examination was normal. On echocardiography, except for small mitral valve regurgitation, intracardiac anatomy abnormalities were not observed. The left ventricular walls were not hypertrophied, the left ventricular internal diastolic dimension was 47.5 mm and remained within the normal range for the patients age and weight (37.5–54.7 mm). Dilatation of the ascending aorta and stenosis of the descending aorta were visible but without a significant poststenotic increase in blood flow velocity. The flow in the abdominal aorta was pulsatile. No prograde blood flow was observed in the left common carotid and in the left subclavian arteries. Spiral computed tomography angiography (SCTA) of heart and aorta (Figs. 1 and 2) showed a thickened thoracic aortic wall (2.7–4.5 mm) and narrowing of its lumen. The aortic measurements and *z* score date, according to Kaiser et al. [23], were performed as presented in Table 1. On the basis of clinical manifestations and angiographic abnormalities, the diagnosis of TA was made.

**Table 1:** The results of SCTA examination

Sites of measurement	Diameter (mm)	*z* score
• Ascending aorta	29	3.87
• First transverse segment	20	
• Second transverse segment	19	
• The isthmic region	14	−1.87
• The minimal diameter of the descending aorta	8	−4.77 (below 3 percentile)
Abnormalities		
• Smooth external outline of the aorta, irregular internal outline, and laminar calcification in the ascending aorta wall		
• Thickened walls of brachiocephalic trunk, right common carotid artery proximal portion (about 7 cm in length), and right subclavian artery proximal portion (about 1 cm in length) with narrowed lumina		
• Complete occlusion of the left common carotid artery and the left subclavian artery		
• Collateral circulation on the thoracic wall and parathyroid vessels		
• Slight thickening of the pulmonary trunk wall		
• Normal abdominal aorta and renal arteries		

Measurements and observed abnormalities

Treatment with prednisone in the initial dose of 60 mg/day (1.3 mg/kg) was introduced together with given orally methotrexate in a dose of 20 mg/m^2^/week. Hypertension was treated using three medications namely: amlodipine (10 mg/day), hydrochlorothiazide (12.5 mg twice a day), and carvedilol (6.25 mg twice a day). After 3 months, the dose of prednisone was gradually tapered to 10 mg/day. The methotrexate has been maintained at the initial dose. The follow-up of this girl until now is about 8 months. Initially the hypertension was not well controlled, so the option of stenting of the descending thoracic aorta had also been considered. But due to the fact that nonspecific markers of inflammation were elevated and this girl had never been treated before, she was qualified for continued medical treatment. At present, under treatment, her blood pressure on the right arm varies between 123/70 and 140/96 mmHg and there is no pressure difference between her right arm and legs. In laboratory tests, C-reactive protein is only slightly elevated 0.67 mg/dl, ESR is 17 mm/h. Moreover, an SCTA examination after a couple of months revealed a similar range of inflammatory changes within the aorta but the thickness of the infiltration had been reduced. The patient is currently under a long-term clinical surveillance by a cardiologist, rheumatologist, nephrologist, and psychologist.

## Discussion

Although our knowledge of TA has considerably improved over the last decade, the etiology and pathogenesis of this disease still remain controversial. It is now assumed that the underlying pathogenesis is inflammatory with unknown etiology. Several etiologic factors have been proposed, including spirochetes, *Mycobacterium tuberculosis*, streptococci, circulating antibodies due to an autoimmune process, and genetic aspects [44]. One hypothesis states that an antigen deposited in vascular walls activates CD4+ T cells, followed by the release of cytokines chemotactic for monocytes. These monocytes are transformed into macrophages that mediate endothelial damage and granuloma formation in the vessel wall. Human studies, suggesting endothelial cell activation, have demonstrated increased expression of intercellular adhesion molecule-1 and vascular cell adhesion molecule-1 in patients with TA [20]. Humoral immunity may also play a role in the pathogenesis. Antimonocyte antibodies and anti-endothelial cell antibodies are present in patients with TA and correlate with disease activity [8]. Genetic susceptibility to TA has been extensively studied. A significant association with HLA B-52 and DR-2 was demonstrated in Japanese patients, but this finding was not confirmed in the western countries [28, 39]. Rarely has TA also been associated with other autoimmune diseases such as glomerulonephritis, systemic lupus erythematosus, juvenile idiopathic arthritis, anterior uveitis, sarcoidosis, seronegative spondyloarthropathy, Crohn’s disease, Wegener’s granulomatosis, Sweet syndrome, and ulcerative colitis, which may indicate immune mechanisms in the pathogenesis [1, 6, 9, 18, 33, 34, 40, 43, 51]. Further investigations are still required to elucidate the pathogenesis of Takayasu arteritis. Considering arterial lesion location, on the basis of angiographic findings, TA is divided into six types [35] (Table 2).

**Table 2:** Angiography-based categories

Type	The vessels involved
Type I	Branches of the aortic arch
Type IIa	Ascending aorta, aortic arch, and its branches
Type IIb	Type IIa region and thoracic descending aorta
Type III	Thoracic descending aorta, abdominal aorta, and/or renal arteries
Type IV	Abdominal aorta and/or renal arteries
Type V	Entire aorta and its branches

There are some differences in the sites affected by inflammatory process among different ethnic groups. Japanese patients with TA have a higher incidence of aortic arch involvement, whereas in patients from Korea, India, and western countries, the abdominal aorta and renal arteries are most frequently affected. However, all patterns of vascular changes have been detected in every country [39]. Clinical manifestations of TA are nonspecific. The clinical course of the disease is divided into an early active inflammatory phase and late chronic phase. The active phase lasts for weeks to months and may have a remitting and relapsing course. It is characterized by systemic disease with symptoms of fever, general malaise, night sweats, loss of appetite, weight loss, headaches, dizziness, arthralgia, skin rashes, etc. The acute phase does not occur in all patients, but constitutional symptoms are often seen in children with TA. It should be highlighted that the correct diagnosis of TA is seldom made in the early phase. Evidence of vessel inflammation such as tenderness along arteries, bruits, and aneurysm may point to the diagnosis of TA [28]. The late chronic phase is the result of arterial stenosis and/or occlusion and ischemia of organs. Its clinical manifestations are varied and related to the location of arterial lesions [28, 39], as presented in Table 3.

**Table 3:** Clinical features of Takayasu arteritis related to ischemia

The vessels involved	Clinical features
1. Aortic branches	Malaise, decreased or absent pulse of upper extremities, dysfunction of upper extremities, headaches, dizziness, vision and orientation disturbances, syncope [25, 48]
2. Aortic arch	Congestive heart failure, aortic valve insufficiency, arterial hypertension [13, 14, 29]
3. Coronary arteries	Ischemic heart disease, myocardial infarction [4, 11]
4. Pulmonary arteries	Chest pain, dyspnea, coughing, hemoptysis, congestive heart failure [22]
5. Abdominal aorta or celiac trunk	Ischemia of the stomach and intestines, abdominal pain, nausea, vomiting [45]
6. Renal arteries	Arterial hypertension, chronic renal failure [5, 7, 15]

It should be remembered that, although TA has been described in patients of all races, reports of Caucasian patients in Europe are infrequent [2]. Therefore, key data concerning the condition, both in adults and in children, come from experience in Asian as well as South African and South American countries. At present, TA in children is diagnosed on the basis of the criteria proposed by European League Against Rheumatism—Pediatric Rheumatology International Trials Organization—Pediatric Rheumatology European Society, Ankara 2008 [41], presented in Table 4.

**Table 4:** The diagnostic criteria for Takayasu arteritis in children

The mandatory criterion
The presence of an angiographic abnormality, i.e., aneurysm/dilatation, narrowing, occlusion or thickened arterial wall not due to fibromuscular dysplasia and shown by conventional angiography, spiral computed tomography angiography or MRA of the aorta or its main branches and pulmonary arteries
In addition to the mandatory criterion, at least one of the five following criteria must be met:
Pulse deficit (lost, decreased, unequal) or claudication (focal muscle pain induced by physical activity)
Discrepancy of four limb systolic blood pressure >10 mmHg difference in any limb
Audible vascular bruits or palpable thrills over large arteries
Arterial hypertension (systolic and/or diastolic blood pressure greater than 95th centile for patient’s age, gender, and height)
Elevation of acute phase reactant (erythrocyte sedimentation rate >20 mm per first hour or serum C-reactive protein of any value above normal according to the local laboratory)

In our patient, all the above-mentioned criteria were met. On the basis of the SCTA examination, TA of type IIb, with pulmonary trunk involvement, was recognized. In our patient case, the initial inflammatory phase of TA did not occur. The onset of her disease was undetectable, and severe systolic arterial hypertension, which was revealed accidentally, was the predominant clinical manifestation.

Pathogenesis of arterial hypertension due to TA is complex, multifactorial, and not fully understood. At present, it is thought to be the result of three mechanisms: (a) mechanical, in which hypertension proximal to narrowed aorta(atypical coarctation) is due to high resistance to cardiac output imposed by narrowing [12]; (b) neural, in which hypertension proximal to narrowed aorta results from aortic arch baroreceptors readjustment and this allows to ensure adequate blood supply to organs distal to narrowed aorta [19]; and (c) hormonal, in which hypertension is caused by renal hypoperfusion due to stenotic lesions of one or both renal arteries or aorta alone [12, 39]. A decrease in elasticity of arterial walls observed in TA may also contribute to the elevation of the blood pressure [37]. In our patient, systolic arterial hypertension seemed to result from narrowing of thoracic aorta, renal ischemia, and probably aortic arch baroreceptors’ hyposensitivity. It should be emphasized that in TA, blood pressure may be over- or underestimated because alternation in wave pulse propagation occurs when cephalad arteries of the aortic arch and both subclavian arteries are affected [48].

The clinical spectrum at presentation of children with Takayasu arteritis differs from that of adults. However, hypertension is the most common sign in both groups [8]. The clinical studies in three large series of pediatric cases from Mexico [10], India [46], and South Africa [16] have revealed that systemic symptoms are seen in a high proportion of children with TA. The usual presenting symptoms are due to hypertension, heart failure, or a neurological event. Claudication, bruit, and a missing pulse in an asymptomatic child are uncommon presentations [28], although those symptoms were present in our patient and they were crucial for establishing the diagnosis.

Suspected TA mandates vascular imaging. While the intra-arterial angiography still remains the standard for diagnosis and evaluation of Takayasu arteritis, it has been largely replaced by computed tomography angiography or magnetic resonance angiography (MRA). These techniques, in addition to visualizing the arterial lumen, may provide valuable information about inflammation in the arterial wall and periarterial structures. This might facilitate the diagnosis of TA at an early stage, in which arterial wall thickening is present and the lumen diameter is preserved [3, 42]. The other investigative modalities, such as gallium-67 radionuclide scanning and positron emission tomography utilizing 18F-FDG, are currently in vogue when evaluating such patients [8, 31], although they are not widely available yet. It should be emphasized that despite the significant advance in noninvasive imaging modalities over past decade, detailed medical history and thorough physical examination still remain important for clinical diagnosis.

Treatment of TA is based on the use of immunosuppressants such as prednisone and/or methotrexate to decrease or eliminate inflammatory activity. About 60% of pediatric patients with TA respond to glucocorticoids. However, as many as 40% relapse on tapering steroids. Alternative therapies such as azathioprine, cyclophosphamide, mycophenolate mofetil, and tacrolimus hydrate are also used in TA, especially for corticosteroid-resistant disease [8, 20, 28, 39]. Hypertension should be treated aggressively often with multidrug regimen, but pediatricians should be warned against ACE inhibitors until renal artery stenosis has been excluded.

In the presence of symptomatic stenotic or occlusive lesions, endovascular revascularization procedures like bypass grafts, patch angioplasty, endarterectomy, percutaneous transluminal angioplasty, or stent placement should be taken into consideration [15, 49, 50]. The status of such treatment is controversial in the literature. Despite providing short-term benefit, endovascular revascularization procedures are associated with a high failure rate in patients with Takayasu arteritis. Published results suggest that these procedures should be undertaken with great care and be reserved for specific indications. Both, surgical and endovascular, treatments become risky and achieve poorer outcomes, if they are undertaken during a period of inflammatory activity [31, 36, 47].

Anti-inflammatory therapy can lead to a dramatic improvement in TA. The 5-year survival rate in adults is as high as 94% [21]. The mortality rate in children, though, is as high as 35% [32]. Increasing knowledge of the pathogenesis of Takayasu arteritis might eventually lead to novel-targeted therapies, such as antitumor necrosis factor agents. Although early diagnosis holds the key to improved outcome, a novel biological approach to treatment might prove more effective and be potentially less toxic than the immunosuppressive regiments used at present [31, 38].
